# Deep learning forecast of rainfall-induced shallow landslides

**DOI:** 10.1038/s41467-023-38135-y

**Published:** 2023-04-28

**Authors:** Alessandro C. Mondini, Fausto Guzzetti, Massimo Melillo

**Affiliations:** 1 Consiglio Nazionale delle Ricerche, Istituto di Ricerca per la Protezione Idrogeologica, Perugia, Italy; 2 Consiglio Nazionale delle Ricerche, Istituto di Matematica Applicata e Tecnologie Informatiche “Enrico Magenes”, Genova, Italy; 3 Presidenza del Consiglio dei Ministri, Dipartimento della Protezione Civile, Rome, Italy

**Keywords:** Natural hazards, Geomorphology

## Abstract

Rainfall triggered landslides occur in all mountain ranges posing threats to people and the environment. Given the projected climate changes, the risk posed by landslides is expected to increase, and the ability to anticipate their occurrence is key for effective risk reduction. Empirical thresholds and physically-based models are used to anticipate the short-term occurrence of rainfall-induced shallow landslides. But, evidence suggests that they may not be effective for operational forecasting over large areas. We propose a deep-learning based strategy to link rainfall to landslide occurrence. We inform and test the system with rainfall and landslide data available for the last 20 years in Italy. Our results indicate that it is possible to anticipate effectively the occurrence of rainfall-induced landslides over large areas, and that their location and timing are controlled primarily by the precipitation, opening to the possibility of operational landslide forecasting based on rainfall measurements and quantitative meteorological forecasts.

## Abstract (web summary)

How much rain does it take to trigger a landslide? This work shows that deep learning can identify the driving forces that can cause rainfall induced landslides, opening up the possibility of forecasting landslide events over large areas

## Introduction

Rain is the leading cause of landslides, globally; but how much rain is needed to trigger a landslide? For decades, this seemingly simple question has resisted attempts to answer it. Recent estimates suggest that landslides occur in about 17.1% of the landmasses, and that about 8.2% of the global population live in landslide prone areas^1^ where people, properties, and the environment are subject to landslide risk^2–4^. Given the projected climate and environmental changes^5^, landslide risk to the population is expected to increase, and particularly the risk posed by rapid-moving, rainfall-induced shallow-landslides^6^. It is therefore not surprising that the interest in landslide forecasting, and for operational geographical landslide early warning systems is increasing in the literature and among decision makers^7–10^.

As with other hazards, the ability to anticipate landslide occurrence is key to the design and the implementation of effective risk reduction strategies^11–13^. In the literature, two approaches are used to predict in space and time the short-term – from a few to tens of hours – occurrence of populations of rainfall-induced shallow landslides – i.e., from one to many landslides caused by one triggering event or by multiple events in a short period^14^ – namely, empirical rainfall thresholds, and physically-based hydrological–slope instability models. Rainfall thresholds define empirically the rainfall conditions that, when reached or exceeded, are likely to result in slope failures [e.g.,^15–18^], whereas physically-based models simulate spatially the hydrological process of the rainfall infiltration trough the ground, and the resulting mechanical consequences for the stability of the terrain [e.g.,^19–22^]. Both approaches assume that the driving force for slope instability is the rainfall – more precisely, the local rainfall history – which acts on surface and sub-surface terrain conditions (e.g., morphometry, geology, surface and sub-surface hydrology, land use and coverage) that together define the propensity – or susceptibility – of the terrain to fail.

In the two approaches, the transient driving force, *R* and the static – at the temporal scale of a landslide triggering rainfall event – terrain settings, *S* are considered to determine where and when landslides are expected to occur in response to a transient input brought on a landscape by a rainfall event. For coupled hydrological–slope instability models the several parameters controlling the surface and subsurface local settings are explicitly considered in the models [e.g.,^19–23^]. When using empirical rainfall thresholds, the role of *S* is considered constructing thresholds for different terrain and environmental settings [e.g.,^24–28^], or (most commonly) it is assumed constant, adopting a single threshold even for large areas with broadly different susceptibility settings [e.g.,^15,27,29,30^]. With a few exceptions [e.g.,^21,31,32^], the physical models are applicable to areas of limited extent, due to their high demand for accurate terrain and environmental data difficult to obtain over large areas^33^. Whereas, empirical thresholds are applicable to all scales, from the local to the global^15,18,30,34^, with different degree of success.

In this work we take a conceptually different approach to the approaches commonly used to anticipate landslides, using rainfall thresholds or coupled hydrological–slope instability models. Our approach is probabilistic, it models the occurrence and the lack of occurrence of landslide events, and measures the uncertainty associated to the forecasts. We show that it is possible to anticipate effectively the occurrence of rainfall-induced landslides over large areas using only hourly rainfall measurements, as the landslide location and timing are controlled primarily by the rainfall. The ability to forecast accurately the possible occurrence of populations of rainfall-induced shallow landslides over large and very large areas without detailed information on the terrain setting makes the approach suitable for operational landslide forecasting at scales raging from the local to the global^9,10,35^.

## Results

**Fig. 1:**
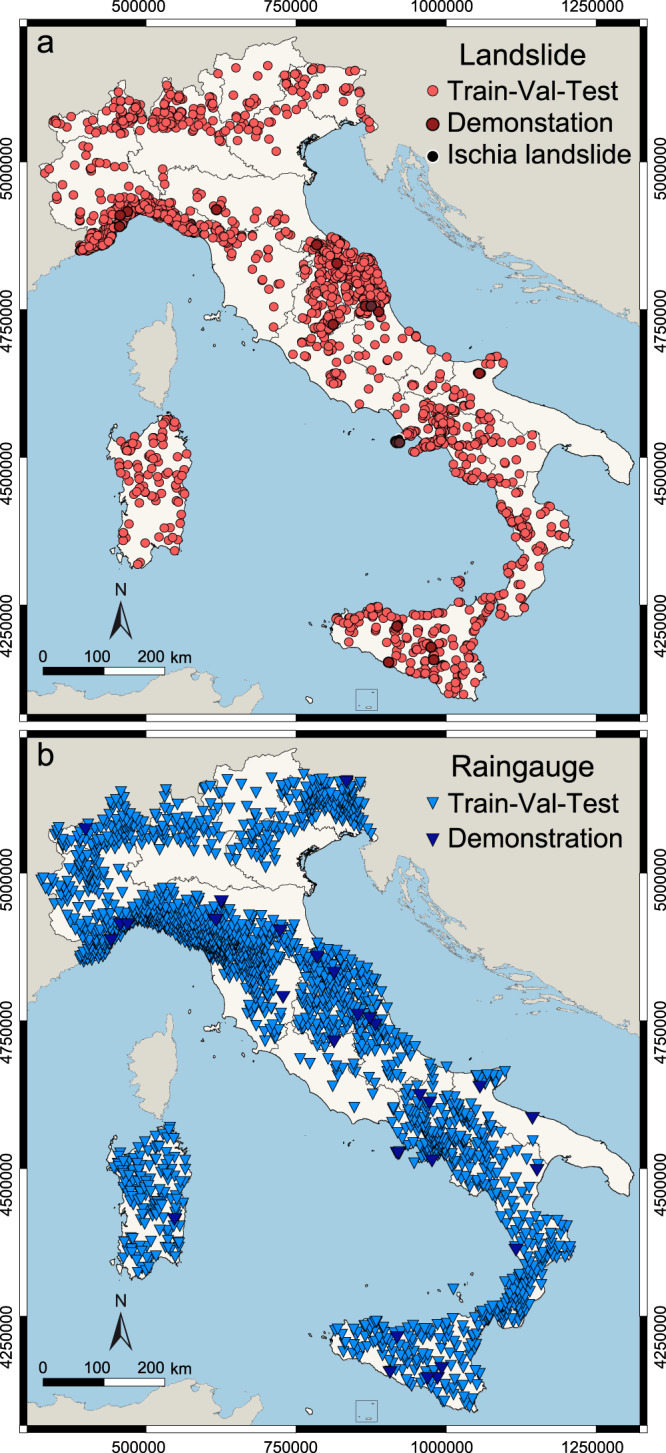
Location of landslide and rain gauge data used in the study. **a** Map shows locations of 2486 rainfall-induced landslides from February 2002 to December 2020 used in the work (red dots), including 14 landslides used to demonstrate the potential operational use of the forecasting system discussed in the Demonstration section (dark red dots). Black dot shows location of the 26 November 2022, Casamicciola Terme landslide, Ischia Island, also used for demonstration. **b** Map shows locations of 2096 rain gauges (blue triangles) for which hourly rainfall records were available to us, of which 29 rain gauges (dark blue triangles) used to demonstrate the potential operational use of the forecasting system. In both maps, geographical and administrative boundaries credits are from the European Environment Agency (EEA) and the Istituto Nazionale di Statistica (ISTAT).

**Fig. 2:**
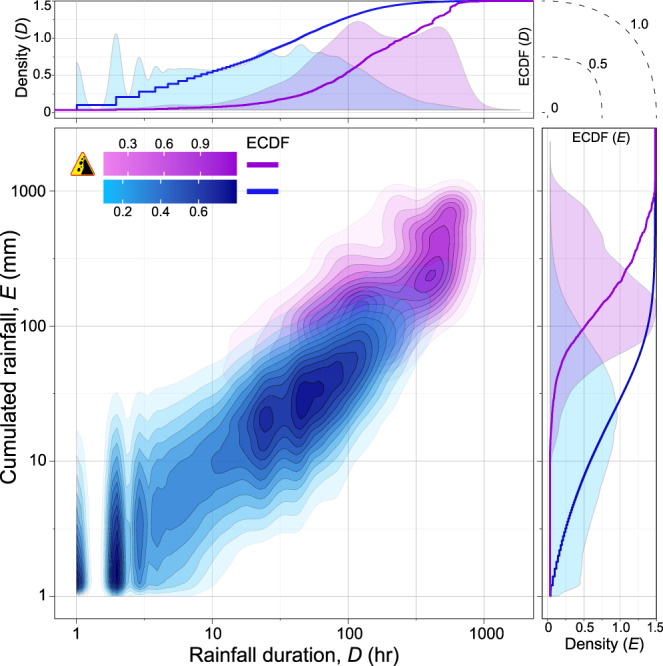
Empirical frequency density distributions of rainfall duration, *D* (h) and hourly cumulated rainfall, *E* (mm) for 780,766 rainfall events in Italy obtained through Two-Dimensional Kernel Density Estimation, KDE2d^79^. Violet shows density for 2472 rainfall events from February 2002 to December 2020 for which the occurrence of at least one landslide is known. Blue shows density for 778,294 rainfall events for which the occurrence of landslide is not known. Upper and right plots show marginal distributions of *D* and *E* (violet and blue areas), and related empirical cumulative distribution functions, ECDF (violet and blue lines) normalised to the number of rainfall events with and without landslides, respectively.

We assume that in a landscape forced by a transient rainfall input – i.e., by a rainfall event, *R* – landslides form, or do not form, on slopes independently from the spatially changing local terrain and environmental conditions, and we propose a deep learning, supervised binary classification approach to distinguish between driving forces able, or not able to trigger rainfall-induced shallow landslides. In mathematical language, the probability of landslide occurrence *F* conditioned by *R*, the rainfall history, and by *S*, the local terrain setting measured by landslide susceptibility, *P*(*F*∣*R*, *S*) becomes *P*(*F*∣*R*) × *c*, with *c* = 1 where landslides can occur, and *c* = 0 elsewhere. We test our hypothesis in Italy, where rainfall-induced shallow landslides are common^27^ and the human consequences severe^36^, and for which a comprehensive catalogue of rainfall events with landslides is available (Fig. 1a), together with rainfall measurements taken by a dense network of 2096 rain gauges (Fig. 1b). Adopting a consolidated technique for the objective reconstruction of rainfall events that can result in landslides^37,38^, we define a rainfall event as a period of nearly continuous rainfall separated from preceding and successive rainfall events by dry periods of no rainfall. Using CTRL-T, the Calculation of Thresholds for Rainfall-induced Landslides Tool software^38^, from the available landslide and rainfall data (Fig. 1) we reconstruct 780,766 rainfall events, of which 2472 (0.3%) with at least one rainfall-induced landslide, and 778,294 (99.7%) with no reported landslides. Figure 2 shows the distributions of the rainfall duration, *D* (in hours) and the corresponding cumulated rainfall, *E* (in mm) computed by CTRL-T, for all the reconstructed rainfall events. This is the information commonly used to define empirical thresholds for possible landslide occurrence^15,18^. Visual inspection of the plot reveals that the distributions of rainfall conditions (*D*, *E*) that did, and did not cause landslides have similar scaling trends, but overlap largely, suggesting the use of an highly non-linear separation model; a task well suited to a neural network modelling approach^39^.

We further assume that a landslide-triggering rainfall event, *R* is composed of two consecutive periods i.e., an antecedent period, *R*_*a*_ that prepares the terrain to fail, followed by a triggering period, *R*_*o*_ when the shear strain in the terrain is reduced and at the end of which the slope fails and a landslide occurs^16, 17,40–42^ i.e., *R* = *R*_*a*_ + *R*_*o*_ (see Supplementary Fig. 1). Rain may continue to fall after the end of *R*_*o*_, but it is irrelevant for landslide occurrence. The lengths of the antecedent and the triggering periods vary, depending e.g., on the rainfall history and the local terrain and environmental settings^16,17,43^. We further assume arbitrarily that in our dataset for Italy the maximum length of the landslide triggering period, *R*_*o*_ is 24 hours. We do not set a limit to the length of the antecedent rainfall period, *R*_*a*_. To represent *R*_*o*_ in the model, we introduce a lag, *ℓ* a continuous sequence of hours, from 1 (*ℓ*_1_) to 24 (*ℓ*_24_).

Each rainfall event, *R* defined by CTRL-T has a starting, *R*_*s*_ and an ending, *R*_*e*_ time, a duration, *D* = [*R*_*s*_; *R*_*e*_], in hours (hr), and a cumulated rainfall ${E}_{[{R}_{s};{R}_{e}]}$, in mm, where the square brackets indicate that the *R*_*s*_ and *R*_*e*_ times are included. In a rainfall event with landslides, the landslide occurrence time is *R*_*f*_, with *R*_*s*_≤*R*_*f*_≤*R*_*e*_, and we assume that (i) the period relevant for landslide initiation is [*R*_*s*_; *R*_*f*_], with a duration *D* = [*R*_*s*_; *R*_*f*_] and cumulated rainfall ${E}_{[{R}_{s};{R}_{f}]}$, (ii) the rainfall period after the landslide, (*R*_*f*_; *R*_*e*_] is irrelevant for the landslide initiation, and (iii) the rainfall in any of the other periods in the rainfall event, [*R*_*s*_; *R*_*f*_) is not sufficient for landslide initiation, where the round brackets indicate that the *R*_*f*_ time is excluded. Ultimately, we assume that all periods in a rainfall event without landslides, regardless of the duration and the corresponding cumulated rainfall, are insufficient for landslide initiation.

### Rainfall variables

We transform the rainfall information shown in Fig. 2 and, for all the rainfall events, we calculate the rainfall duration—cumulated event rainfall pairs for different periods, with and without landslides, in three steps discussed in detail in the Methods section, and we construct two subsets of rainfall data points associated, Y and not associated, X to landslides, which together represent our modelling dataset, with the following rainfall explanatory variables, *D*_*a*_, *E*_*a*_, *D*_*o*_ = *D*_*ℓ*_, and *E*_*o*_, for the antecedent (*D*_*a*_, *E*_*a*_) and the triggering (*D*_*o*_, *E*_*o*_) rainfall periods.

For our modelling, we first extract randomly from the entire dataset, {X + Y}, 15 rainfall events with landslides and 14 rainfall events without landslides to form subset Z (dark blue symbols in Fig. 1) which we will use to demonstrate the system. Next, adopting a train–valid–test data segmentation scheme^44^, we construct three modelling subsets for bagging^45^. For each lag, *ℓ* i.e., for each continuous sequence of hours from 1 (*ℓ*_1_) to 24 (*ℓ*_24_), we split randomly 100 times the remaining rainfall data points with landslides—from 2472 for *ℓ*_1_ to 2092 for *ℓ*_24_—into two subsets, with (i) ≈ 80% of the data points, to which we add the same number of randomly selected data points without landslides, to obtain subsets ${{\mathsf{TV}}}_{i}^{\ell }$, with *i* ranging from 1 to 100, for model training & validation, and (ii) the remaining ≈ 20% of the data points with landslides, and all the remaining data points without landslides, more than 99.99% of all data points, to form subsets ${{\mathsf{W}}}_{i}^{\ell }$ for model testing. We further split randomly subsets ${{\mathsf{TV}}}_{i}^{\ell }$ to obtain (iii) the training subsets, ${{\mathsf{T}}}_{i}^{\ell }$ having ≈ 64% of the data points, and (iv) the validation subsets, ${{\mathsf{V}}}_{i}^{\ell }$ with ≈ 16% of the data points.

### Model structure and parametrization

**Fig. 3:**
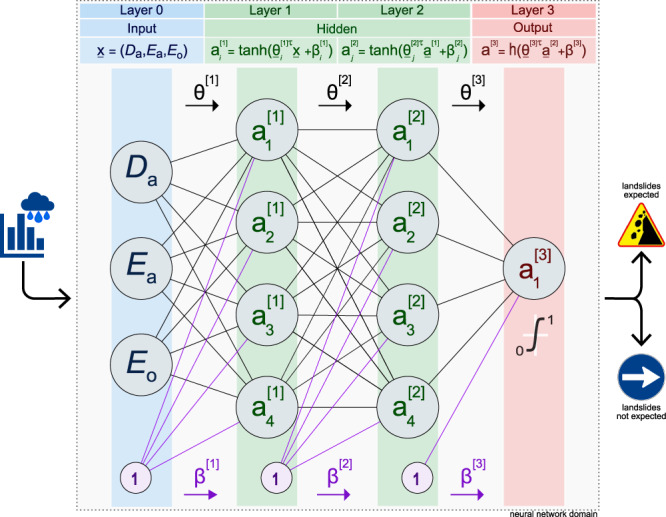
Neural network geometry for every model ${{{{{{{{\mathcal{M}}}}}}}}}_{i}$. Grey circles are neurons arranged in four layers. Blue, input layer 0; green, hidden layers 1 & 2; red, output layer 3. Neurons in the hidden (green) layers are activated by a tanh function. Output (red) layer is activated by a sigmoid function h. ${{{{{{{{\rm{a}}}}}}}}}_{n}^{k}$ is neuron *n* in the *k* layer, ${\beta }_{n}^{[k]}$ in *β*^[*k*]^ is the bias added to the *n* neuron in the *k* layer, and ${\underline{\theta }}_{n}^{k}$ in *θ*^[*k*]^ is the weight array of the neurons in the *k* − 1 layer in the *n* neuron of the *k* layer. *τ* stands for transpose.

Not having sufficient information on the relative role—and hence, of the most appropriate duration—of the antecedent, *R*_*a*_ and the triggering, *R*_*o*_ rainfall periods for landslide initiation^16,17,46,47^, and given the large variability of the rainfall conditions (i.e., *R*_*a*_ and *R*_*o*_) that can result in landslides in Italy^27^, we chose to prepare independent ensembles of models for each lag, *ℓ* where *ℓ* represents different possible rainfall triggering periods, *R*_*o*_. For this reason, we use three of the four explanatory (independent) variables in the modelling dataset i.e., *D*_*a*_, *E*_*a*_, and *E*_*o*_, since *D*_*o*_ = *D*_*ℓ*_ is constant for each lag, and would not contribute to the separation of the rainfall conditions that can, and cannot trigger landslides. We opt for a fully connected neural network with one input layer to ingest the three explanatory variables, two hidden layers with four neurons each, and one output layer with a single neuron (Fig. 3). The model (${{{{{{{\mathcal{M}}}}}}}}$) provides the probability of landslide occurrence, given the three explanatory variables (*D*_*a*_, *E*_*a*_, and *E*_*o*_), and a set of 32 weights, ***θ*** and 9 biases, ***β*** connecting the neurons obtained in the model calibration phase, ${P}_{{{{{{{{\mathcal{M}}}}}}}}}(1|{{{{{{{\bf{x}}}}}}}},\, {{{{{{{\boldsymbol{\theta }}}}}}}},\, {{{{{{{\boldsymbol{\beta }}}}}}}})$^48,49^.

### Model training and validation

For each of the 24 lags, we train and validate 100 different models using the 100 pairs of training, ${{\mathsf{T}}}_{i}^{\ell }$ and validation, ${{\mathsf{V}}}_{i}^{\ell }$ subsets, with *i* ranging from 1 to 100 (bagging ensemble^45^). This allows to evaluate the model ensemble accuracy and variability. We estimate the training and the validation accuracy of each model, ${{{{{{{{\mathcal{M}}}}}}}}}_{i}$ in the training phase as the frequency with which the model prediction matches the true value in the training, T^*ℓ*^ and in the validation, V^*ℓ*^ subsets, ≈ 64% and ≈ 16% of all the rainfall events, nearly 1460 and 365 rainfall events with landslides. Assuming an a priori probability threshold to separate the rainfall events predicted as having, *p* > 0.5 from those predicted as not having, *p* ≤ 0.5 landslides, we obtain accuracies in the range from 84.8% to 78.3%, for both the training and the validation accuracies, with a tendency to stabilise the accuracy for longer lags. The similar model performance for the ${{\mathsf{T}}}_{i}^{\ell }$ and the ${{\mathsf{V}}}_{i}^{\ell }$ subsets for all *i* and *ℓ* reveals the lack of overfitting, and a good generalisation capacity of the single models ${{{{{{{{\mathcal{M}}}}}}}}}_{i}$^49^ (see Supplementary Figure 2a, b, c).

### Model testing

To test the performances of our model bagging ensembles, ${{{{{{{{\mathcal{B}}}}}}}}}^{\ell }$ we use W^*ℓ*^. To handle the severe class imbalance inherent in the W^*ℓ*^ subsets, with a ratio of data points with & without landslides of ≈ 1/100,000, we arbitrarily determine the probability threshold for each model as the best trade-off between the model sensitivity i.e., the true positive rate TPR = TP/(TP+FN), and specificity i.e., the false positive rate, FPR = FP/(FP+TP) in a typical Receiver Operating Characteristic (ROC) curve^50^.

**Fig. 4:**
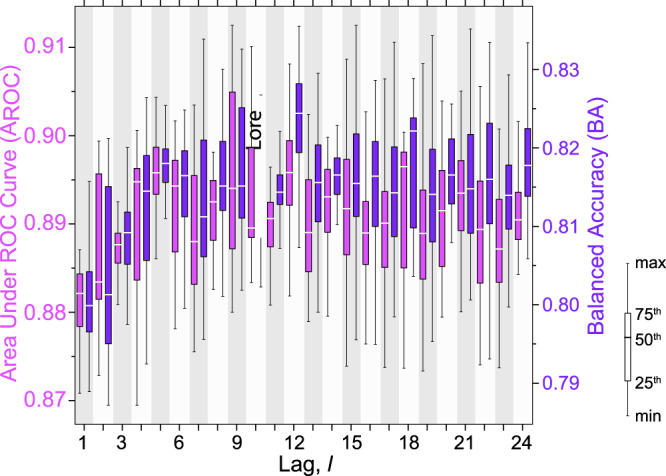
Box-and-whisker plots showing performance of the 2400 ${{{{{{{{\mathcal{M}}}}}}}}}_{i}^{\ell }$ models of set ${{{{{{{\mathcal{O}}}}}}}}$, in the model testing phase. For each lag, *ℓ* (x-axis), purple boxplots show variability of the *A*_*ROC*_ of the 100 ${{{{{{{{\mathcal{M}}}}}}}}}^{\ell }$ models (left y-axis), and violet boxplots show Balanced Accuracy, BA^51^ (right y-axis). White horizontal lines show median value for each boxplot. Boxplot legend: min and max are minimum and maximum values; 25^*th*^, 50^*th*^ (median), and 75^*th*^ are percentiles.

For all lags, the median of the area under the ROC curves, *A*_*ROC*_ is large ( ≈ 0.92 to ≈ 0.88, Fig. 4), with larger ranges for model ensembles with higher medians and little or no skewness. We note that the single models are all far from a random guess (*A*_*ROC*_ = 0.50), and that the model ensembles perform more uniformly for long (*ℓ* ≥ 12) than for short (*ℓ* ≤ 6) triggering periods. The general behaviour is confirmed by the Balanced Accuracy, BA a good performance metric for imbalanced data sets averaging sensitivity and specificity^51^, with median values from ≈ 0.80 to ≈ 0.82, with little variability and very low skewness (Fig. 4). The large values of the two metrics are evidence of the high performances of the single models, ${{{{{{{{\mathcal{M}}}}}}}}}_{i}$ and of the ensemble sets, ${{{{{{{{\mathcal{B}}}}}}}}}^{\ell }$ also for highly unbalanced datasets; a typical case for landslide forecasting where the number of rainfall events with landslides is much smaller that the number of rainfall events without landslides^28,52,53^. We hypothesize that the (slightly) larger volatility of the performances of the ensemble sets for shorter triggering periods is due to local effects in the landslide occurrence process, with shorter rainfall triggering periods better capturing local instability conditions.

### Demonstration

We now demonstrate the potential operational use of our set of bagging ensemble models, ${{{{{{{\mathcal{O}}}}}}}}$ combined through a simple voting scheme^45, 54^. For the purpose, we exploit the Z record set encompassing 14 + 1 (the Casamicciola Terme landslide) rainfall events with landslides and 14 rainfall events without landslides in different physiographic settings in Italy (Fig. 1). We assign to the events in the Z record set the rainfall – in this case – predicting variables *D*_*a*_, *E*_*a*_, and *E*_*o*_, for all 24 lags, from *ℓ*_1_ to *ℓ*_24_. Next, for each *ℓ*, we enter the three predicting variables in the corresponding trained and validated model ensembles to obtain the related probabilities of landslide occurrence, that we transform into crisp (i.e., “landslide" or “no landslide") classifications using the probability thresholds determined in the model test phase.

We then combine the 100 individual forecasts in each lag by voting^45,54^, where the final vote for each lag is given by *V*_*ℓ*_ = *argmax* (#"landslides") / (#"no landslides"), and we estimate the variance of *V*_*ℓ*_ as *σ*^2^ = (#"landslides”/100) × (#"no landslides”/100), a measure of the dispersion of the votes of the single models, ${{{{{{{{\mathcal{M}}}}}}}}}_{i}^{\ell }$ in the bagging ensemble sets, ${{{{{{{{\mathcal{B}}}}}}}}}^{\ell }$. Then, using the same argmax criterion, we merge the 24 combined votes, one for each lag, into a single, aggregated vote, $\hat{V}$—our forecast—on the possible occurrence of a landslide during the rainfall event.

**Fig. 5:**
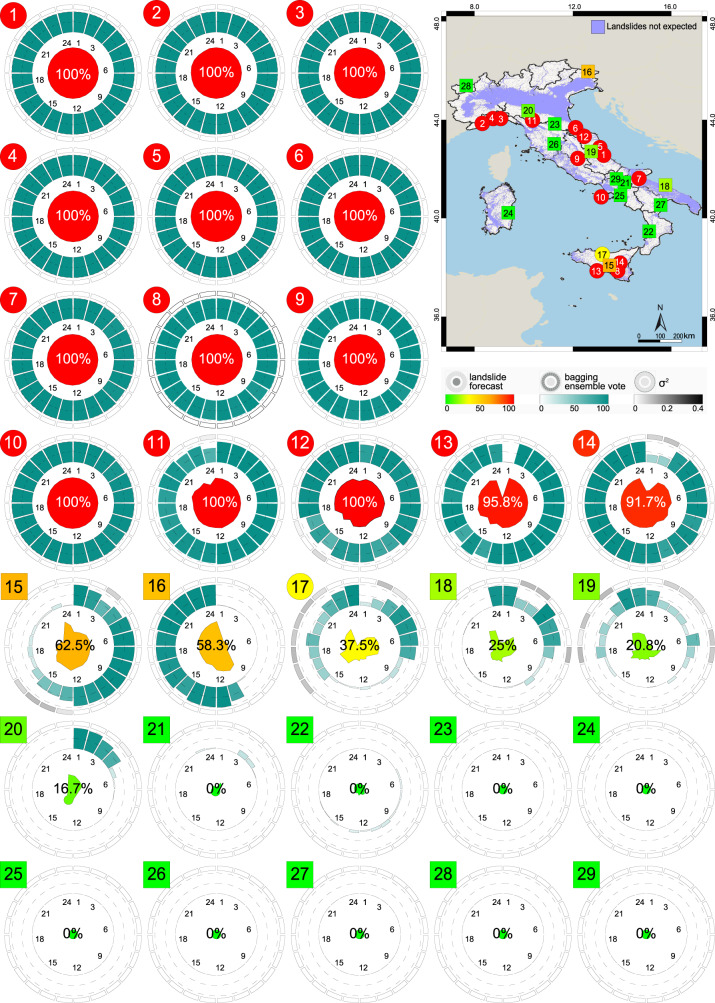
Demonstration of the forecasting system based on ${{{{{{{\mathcal{O}}}}}}}}$, our set of 2400 models. Symbols in the map and near the circular plots show 15 (14 + 1 i.e., #10, the 26/11/2022 Casamicciola Terme landslide) rainfall events with landslides (circle), and 14 rainfall events without landslides (square). Map shows locations of 29 rain gauges for which the system is demonstrated. For location of the events see Fig. 1. Colours show voting scores, from 0 (green) to 24 (red). Violet shows areas where, according to Marchesini and co-workers^80^, landslides are not expected based on morphometry i.e., *c* = 0 in *P*(*F*∣*R*) × *c*. Circular plots show voting statistics for the 29 rainfall events, for each lag, from *ℓ*_1_ to *ℓ*_24_, in clockwise order. Inner spider plots show the scores, with the colour and the size of the coloured area giving the percentage of lags that predicted landslide occurrence, from green (0%), to yellow (50%), to red (100%), also given by the central figure, in percentage. Radial bars show landslide occurrence voting scores, from white (0) to teal (100), of each ${{{{{{{\mathcal{{B}}}}}}}^{\ell }}}$, for each lag, *ℓ*. Outer plots give the variance (*σ*^2^) of the 100 votes, from nil (white) to large (dark grey). In the map, geographical and administrative boundaries credits are from the European Environment Agency (EEA) and the Istituto Nazionale di Statistica (ISTAT).

For the 29 demonstration rainfall events in the Z set, the map in Fig. 5 portrays the aggregated vote, $\hat{V}$ for rainfall events having (circles) and not having (squares) landslides, coloured from green—where none of the 24 combined votes are for landslide occurrence—to red—where all the 24 combined votes are for landslide occurrence. In Fig. 5 the individual circular charts summarize statistics for the 24 model bagging ensemble sets, ${{{{{{{{\mathcal{B}}}}}}}}}^{\ell }$ shown in clockwise order from *ℓ*_1_ to *ℓ*_24_. The inner spider plots show the proportion of the 24 lags voting for (red) and against (green) landslide occurrence. The central plots show the percentage of votes for landslide occurrence in the 100 models of each ${{{{{{{{\mathcal{B}}}}}}}}}^{\ell }$, with their variance, *σ*^2^ shown by the outer plots in greyscale.

Visual inspection of Fig. 5 does not reveal any geographical or other biases in the model performance. This confirms that the model set, ${{{{{{{\mathcal{O}}}}}}}}$ is a good predictor of the possible occurrence of rainfall-induced shallow landslides in Italy. Analysis of the 29 circular charts in Fig. 5 reveals that 14 (out of 15, 93.3%) of the rainfall events with landslides (red dots) were correctly voted (i.e., true positives, TP in Z) with an agreement among the ensemble sets, ${{{{{{{{\mathcal{B}}}}}}}}}^{\ell }$ larger than 91%, and that the single incorrectly voted (i.e., false positive, FP in Z) event (#17) has ≈ 63% of incorrect votes. Similarly, 12 (out of 14, 85.7%) of the rainfall events without landslides (green squares) were voted correctly (i.e., true negatives, TN) with an agreement ≥ 70% among the ensemble sets ${{{{{{{{\mathcal{B}}}}}}}}}^{\ell }$. The two incorrectly voted rainfall events, (#15, #16) have about 62.5% and 58% of incorrect votes. For the almost balanced set of events Z, we obtain a Cohen’s kappa, *κ* = 0.79^55^, and a *F*_1_ score = 0.90^56^. We note that the true positives are better represented than the true negatives, and that the true negatives show a larger uncertainty than the true positives. This was expected, given the much larger number of rainfall events without landslides.

Overall, in Fig. 5, the map and the associated charts, portray all the information that a landslide forecaster in an operational centre, or an automatic landslide early warning system (LEWS)^7–10^, can use to inform with proper advisories timely actions to elicit appropriate risk mitigation responses^10, 12,13,28^. The forecast can be readily updated whenever new rainfall measurements, nowcasts or forecasts become available, to anticipate, in space and time, the evolution and the possible effects of a rainfall event in an area, assessing its expected ability to generate landslides based on the past and the expected rainfall history.

## Discussion

Our approach to anticipate where and when in a landscape forced by a rainfall event rainfall-induced shallow landslides are expected is different from the approaches adopted by empirical rainfall thresholds and by physically-based hydrological–slope instability models. Typically, empirical rainfall thresholds use only information on rainfall events that have resulted in landslides, and disregard the events that have not resulted in landslides. Further, threshold models do not consider the dynamics of the driving force i.e., the rainfall history that leads to slope failures. Physically based models simulate the hydrological and mechanical processes acting in the slopes forced by a rainfall event, but at any given time and location the model outputs depend solely on the local conditions, as the models do not consider explicitly the rainfall history, and its spatial and temporal consequences. Our approach overcomes these limitations considering all the rainfall conditions that have, and have not resulted in landslides, and the event rainfall history.

With our modelling exercise, we showed that exploiting a relatively simple data-driven, deep-learning based approach (Fig. 3), it is possible to forecast with very good performances (e.g., from *A*_*ROC*_ ≥ 0.87 for *ℓ*_1_ to *A*_*ROC*_ ≥ 0.91 for *ℓ*_9_, Fig. 4), and over large areas (in our case, ≈ 301,000 *km*^2^), the possible occurrence of (future) rainfall-induced shallow landslides using predictors obtained from a record of hourly rainfall measurements, and information on the (past) occurrence, and lack of occurrence, of event-triggered shallow landslides, without the need for detailed terrain and environmental information.

Albeit the scope of our data-driven framework was to make reliable forecasts of the possible occurrence of rainfall-induced shallow landslides and, hence, a direct interpretability of the model insight in terms of physical relationships between the input variables (i.e., the rainfall history, *R*) and the forecasts (i.e., the anticipation of the landslide occurrence) is not required^39^, we maintain that the empirical evidence suggests two general results of geomorphological and operational interest.

From a geomorphological perspective, we infer that in a landscape forced by a transient rainfall field, the main factor controlling the initiation of shallow landslides is the rainfall i.e., the landslide event driving force. The finding suggests that in the areas of a landscape where landslides can occur due to the local terrain settings^1^, landslides will occur where and when a set of—locally often unknown—rainfall conditions are reached or exceeded^15^. The local terrain and environmental conditions, including e.g., the morphometric, geological, hydrological, structural, land use and land cover conditions, control the location of the landslide initiation points at a much finer scale than the characteristic scale of the landscape considered in this work. We stress that the local terrain and environmental conditions are difficult and expensive to obtain with the adequate accuracy over large and very large areas. With a few exceptions^57^, this limits their use to specific cases and to areas of limited extent^58,59^.

From an operational perspective, we note that the forecasting performances are very good, considering the difficulty of the task^14^, and the paucity of the data. Our results open to the possibility that geographical landslide early warning systems (LEWSs)^7–10^ working at scales from the local to the global^35,60^, can predict the occurrence of populations of rainfall-induced landslides using exclusively rainfall measurements and quantitative rainfall forecasts, overcoming the need for detailed, expensive, and difficult to obtain and update terrain and environmental information used to construct landslide susceptibility^33^ and hazard^61^ models. The finding suggests that the operational prediction of populations of rainfall-induced landslides can become part of standard weather forecasts, as anticipated nearly half a century ago by Russell H. Campbell^46^, provided that reliable quantitative rainfall forecasts or nowcasts e.g., through meteorological radars, are available.

We expect our forecast model set, ${{{{{{{\mathcal{O}}}}}}}}$ to perform well in the same general area where the rainfall and the landslide information was available to construct the model i.e., in Italy, and in other geographical areas characterised by similar meteorological and climatic regimes; firstly, in the landscapes inside and surrounding the Mediterranean basin.

Ultimately, we stress that our approach is functional i.e., it depends on the available data, and it assumes the stationarity of the rainfall and the landslide records, which are not guaranteed over long periods and where climate, environmental, and geological changes are large^6^. Should the changes be significant, the model performance will need to be re-assessed, the model set re-calibrated, or the neural architecture redesigned entirely.

## Methods

### Rainfall and landslide data

We use two data sources. We obtain information on the occurrence of rainfall-induced (mostly shallow) landslides in Italy from an updated version of the catalogue prepared by Peruccacci and her coworkers^27^ built searching multiple sources of information, including national, regional, and local newspapers, and event and fire fighter reports. Our catalogue lists 2486 landslides from February 2002 to December 2020, and the 26 November 2022 Casamicciola Terme landslide, in all physiographical areas where landslides are expected in Italy (Fig. 1a). In the catalogue, landslides are first-time (new) failures, first-time failures occurred inside pre-existing landslides, or partial or total reactivations of pre-existing landslides. Temporal and geographical accuracies were attributed to each landslide in the catalogue^27,62^. We select landslides with a temporal accuracy of one hour, consistent with our modelling of the rainfall information in one-hour lag periods, and with a minimum geographical accuracy of ≈ 10 km; with 98.1% of the landslides within ≈ 1.8 km, and 25.7% of the landslides located exactly.

We obtain hourly rainfall measurements from a national network of 2096 automatically recording rain gauges operated by Regional and Provincial governments in Italy (Fig. 1b). This is an average of one rain gauge every ≈ 144.5 *km*^2^—a high gauge density^63^, with an average gauge spacing of ≈ 12.0 km. Overall, the rainfall dataset contains more than 300 million records.

Using CTRL-T, the Calculation of Thresholds for Rainfall-induced Landslides Tool software^38^, we reconstruct 780,766 rainfall events, of which 2472 (0.3%) with at least one rainfall-induced landslide, and 778,292 (99.7%) with no reported landslides.

To separate the events in the rainfall record, we use a 48-h period without rainfall for the dry season [June to September], and a 96-h dry period for the wet season [October to May]^27,37^. The rainfall events without landslides have 1 hr ≤ *D* ≤ 2150 hr ( ≈ 90 days), and cumulated rainfall 1 mm ≤ *E* ≤ 2070 mm, whereas the rainfall events with landslides have 1 hr ≤ *D* ≤ 1537 hr ( ≈ 64 days), and cumulated rainfall 3 mm ≤ *E* ≤ 1330 mm (Fig. 2).

### Landslide and rain gauges association

Following Peruccacci and her coworkers^64^, we assign each landslide in the catalogue to a single rain gauge considering the topographic distance and the elevation difference between the landslide and the rain gauge, and the local morphological setting. We could instead interpolate the rainfall history at the landslide site using the near available rain gauges. Given the uncertainty inherent in the landslide location, this would not result in any significant improvement in the definition of the rainfall history at the landslide site^65^.

### Modelling datasets and rainfall variables

To prepare the modelling dataset {X + Y} of rainfall data points associated, Y and not associated, X to landslides, we adopt the following procedure.

To construct subset X, for each rainfall event without landslides:

- Step 1. For each *ℓ*, where *ℓ* is a continuous sequence of hours from 1 (*ℓ*_1_) to 24 (*ℓ*_24_) hours, we compute the duration of the antecedent, *D*_*a*_ = [*R*_*s*_; *R*_*e*−*ℓ*_] and the triggering, *D*_*o*_ = *D*_*ℓ*_ periods, and the corresponding cumulated rainfall, *E*_*a*_ and *E*_*o*_, obtaining, for each *ℓ*, *D*_*a*_, *E*_*a*_, *D*_*o*_ = *D*_*ℓ*_, and *E*_*o*_, which we enlist in subset X of data points not associated to a landslide.
- Step 2. We move artificially the end of the rainfall event one hour backwards i.e., [*R*_*s*_; *R*_*e*−1*hr*_], and we repeat Step 1, for all *ℓ*.
- Step 3. We set *R*_*e*_ = *R*_*e*−1*hr*_, and we repeat Step 2 until *D*_*a*_ = 1 h.

Similarly, to construct subset Y, for each rainfall event with landslides:

- Step 4. For each *ℓ*, from (*ℓ*_1_) to 24 (*ℓ*_24_) hours, we compute the duration of the antecedent, *D*_*a*_ = [*R*_*s*_; *R*_*f*−*ℓ*_] and the triggering, *D*_*o*_ = *D*_*ℓ*_ periods, and the corresponding cumulated rainfall, *E*_*a*_ and *E*_*o*_, obtaining, for each *ℓ*, *D*_*a*_, *E*_*a*_, *D*_*o*_ = *D*_*ℓ*_, and *E*_*o*_, which this time we enlist to the subset Y of data points associated to a landslide. We disregard the rainfall period after the landslide, (*R*_*f*_; *R*_*e*_] considered irrelevant for the landslide initiation.
- Step 5. We move artificially the time of the landslide occurrence one hour backwards i.e., [*R*_*s*_; *R*_*f*−1*hr*_], and we repeat Step 4, for all *ℓ*. This time, we assign the computed *D*_*a*_, *E*_*a*_, *D*_*o*_ = *D*_*ℓ*_, and *E*_*o*_, to the subset X of data points not associated to a landslide, because a landslide did not occur in the period [*R*_*s*_; *R*_*f*−1*hr*_].
- Step 6. We set *R*_*e*_ = *R*_*e*−1*hr*_, and we repeat Step 5 until *D*_*a*_ = 1 hour.

### Net configuration and settings

To select the final network architecture, we tested 4 × 4, 6 × 6, 8 × 8, 8 × 4 × 8, and 8 × 8 × 8 network geometries for lags *ℓ*_1_, *ℓ*_12_, and *ℓ*_24_. Since complex or deep neural architectures are often an overkill where data are scarce^49^, we select the simplest 4 × 4 configuration and we keep it fixed (Fig. 3). For network optimisation, we randomly initialise the 32 weights of each model, ***θ*** with a normal distribution with mean, *μ* = 0 and standard deviation, *σ* = 0.1, and we set the biases to zero, ***β*** = 0. All layers in the network are regularised using the L2 regularization penalty factor^66^, a weight decay, *δ* = 0.001, and a dropout, *γ* = 0.25^67^, to mitigate overfitting^49,68^. All neurons are activated through an hyperbolic tangent function, tanh except the last neuron which is activated by a sigmoid function, h. Assuming landslide occurrence obeys a Bernoulli distribution, we build the model using the binary cross-entropy loss function^69^, 0 ≤ *p*(*y* = *”yes”*) ≤ 1, and the Adaptive Moment Estimation method (Adam)^34,70^ with an inverse time decay for the learning rate initially set to 1*e*^−4^, a decay rate, *δ* = 0.05 every 100 update steps, *β*_1_ = 0.9, *β*_2_ = 0.999, and $\acute{\epsilon }=1{e}^{-7}$^70^. We set the training phase using a batch size of 32, and a maximum number of epochs of 200,000, including an early stopping over the loss function with 1000 steps of patience, and a minimum decay rate *δ* = 0.00001^71^.

### Overfitting

All the loss and accuracy curves for the training and the validation sets of the model set ${{{{{{{\mathcal{O}}}}}}}}$ converge numerically to very similar values (see Supplementary Figure 2a, b, c). Occasionally, and more frequently for lags ≤ *ℓ*_5_, the accuracy curve for the T set is ≈ 1% to 2% higher than the curve for the V^*ℓ*^ set (see Supplementary Figure 2c), or vice-versa, indicating an excessive regularisation, or a poor representativeness of the randomly selected training sets. We conclude that the preventive exclusion from ${{{{{{{\mathcal{O}}}}}}}}$ of the models prepared with low representative training sets, or a lag-dependent fine tuning of the network geometry and of the related parameters, might lead to better generalization capacity of the single ${{{{{{{{\mathcal{M}}}}}}}}}^{\ell }$ models in ${{{{{{{\mathcal{O}}}}}}}}$. However, such fine tuning is out of the scope of this work.

### Alternative data segmentation strategies

To construct our network, we opted for a classical train–valid–test random data segmentation scheme^44^, and we generated balanced – i.e., having the same number of landslide and no landslide events – train, T^*ℓ*^ and validation, V^*ℓ*^ sets for the network tuning phase. In reality, in many cases a rainfall event does not trigger landslides, resulting in a large unbalance. We cope with the problem using unbalanced sets, the W^*ℓ*^ sets listing ≈ 450 rainfall events with landslides (0.00001%) and ≈ 40 million rainfall events without landslides (99.99999%), a 1/1,000,000 ratio, for testing the network. The W^*ℓ*^ sets are fixed for each *ℓ* to allow for the comparison of the ${{{{{{{{\mathcal{B}}}}}}}}}^{\ell }$ models. We acknowledge that an increase of the penalty for probabilistic false negatives during the training phase^69^, other data splitting strategies with different “landslide” / “no-landslide” ratios, or other evaluating metrics could be used to consider the inherent imbalance of the modelled process.

### Alternative classification methods

Other methods exist to discriminate rainfall conditions that can and cannot trigger landslides. The most promising include support vector machines (SVMs) and long-short term memory (LSTM) convolutional neural networks. SVMs are known for their performance with small data sets when properly tuned, and they can work with the same data structure used in our experiment. Hence, they represent a clear alternative to our neural network approach. In fact, we have no a priori justification for preferring our approach to SVMs, except that in the initial phase of research, non-linear SVMs performed slightly less well than our neural networks. Convolutional LSTM neural networks deal effectively with time series in which an effect (e.g., the occurrence of a landslide) depends on a sequence of feedbacks (e.g., the rainfall record). Convolution makes it possible to exploit spatial rain fields to improve the spatial representation of the forecast. As a limitation for our case study, convolutional LSTM neural networks require further research to work with landslide events associated with gridded rainfall data in the training phase, and a different definition of a rainfall event.

### Uncertainties

Our experiment suffers from aleatory and epistemic uncertainty^72^. The aleatory uncertainty is typical of the many physical processes that control the landslide initiation, and cannot be cancelled. Epistemic uncertainty affects the landslide and the rainfall data, and the neural network model structure and parameters. Our landslide catalogue is relatively large and it is accurate, but like any other unsystematic, non-instrumental source of information, it is incomplete and the level of completeness remains unknown. The landslide catalogue covers 19 years. In this period, not all the areas where landslides can occur in Italy were affected by rainfall events, and specifically events that could trigger landslides. A longer period would guarantee a better coverage, but would bring additional uncertainty due to environmental and climate changes that affect the rate of landslide occurrence^42,73^.

In the original catalogue^27^, the lack of accurate information on the exact time of occurrence of the landslides – a notoriously difficult information to obtain^15,74,75^ – limits the ability to reconstruct the rainfall triggering conditions, reducing the number of events that can be used to train the system.

As a result, some of the model false negatives are due to lack of information on landslide occurrence, in particular for rainfall records which are apparently indistinguishable from records that have resulted in shallow landslides for some lags. In these cases, the forecasts show large uncertainties, and we maintain that use of different lags helps reducing possible errors.

We set the maximum length of the landslide triggering period, *R*_*o*_ in a rainfall event to 24 h, and we model it with different lag periods, *ℓ*. We acknowledge that the selection was arbitrary, but we maintain it is reasonable for the scope of the work. Given the tendency of the forecasting capability of the model sets towards similar values for longer lag periods (Fig. 4), selecting a longer maximum *R*_*o*_ would not increase substantially the prediction performance, and would add epistemic and aleatory uncertainty to the results.

Epistemic uncertainty also affects the rainfall measurements and, hence, the reconstruction of the rainfall history, and the true (and unknown) rainfall characteristics that trigger, or do not trigger landslides during a rainfall event. These are typical of all attempts to measure rainfall, and to model and anticipate landslide occurrence using rainfall measurements^65,76–78^.

Epistemic uncertainty associated to the neural network model set are related to the selection of the type of the neural network, the geometry of the net (e.g., number of layers, neurons), and the selection of the model hyperparameters (e.g., *β*_1_, *β*_2_, *δ*, *γ*). Use of large bagging ensembles with 100 independent model repetitions, albeit not reducing the uncertainty, has contributed to quantify it, or part of it. The variance of the votes for the models ensemble, ${{{{{{{{\mathcal{B}}}}}}}}}^{\ell }$ measures the models agreement, and it provides a measure of the confidence with which the final vote should be considered; a low (high) variance is the result of a large (reduced) agreement among the models in each lag, and gives a high (low) confidence in the forecast. Forecast users should check the cases with a high variance (e.g., cases #15, #16, and #17 in Fig. 5). We maintain they are a minority.

### Alternative variables

Our experiment revealed that variables obtained from standard hourly rainfall records are sufficient to anticipate accurately the possible occurrence of future landslides. Regardless, the inclusion of additional explanatory variables could further improve the overall model forecast accuracy, provided the variables are collected and significant at the scale of the analysis. Terrain morphology, lithology, soil type, land cover, land use, and soil moisture are candidates, and their use in a different, more complex neural network might capture more complex associations, reducing in particular the false positives due to similarity of the rainfall records that resulted and did not result in landslides. Alternatively, one could use susceptibility as a lumped measure of the local terrain conditions favourable or not favourable to landslide occurrence^33^. However, given the already high classification performance of our simple, 4 × 4 neural network, it is not certain that use of additional explanatory variables will increase the classification performance, reducing the misattributed cases, necessarily. The new variables may not be sufficiently accurate to capture the landslide initiation process adding uncertainty, and considering them may not result in better performances^72^. When using variables that change over time (e.g., seasonal variables) or collected over a period (e.g., digital terrain models covering large areas), the temporal relation between the thematic, terrain, and landslide information should be considered, as it may add uncertainty and reduce model performance.

### Software and hardware details

The model is written in Tensorflow 2.3.0 (https://www.tensorflow.org/) with the support of Python 3.8 (https://www.python.org/), Keras 1.1.2 (https://keras.io/), Scikit-learn 0.24.1 (https://scikit-learn.org/), Numpy 1.21.2 (https://numpy.org/), and Pandas – Python Data Analysis Library 1.2.3. (https://pandas.pydata.org) in Ubuntu® 20.04 (https://ubuntu.com/).

Time to tune a single model varied depending on the time to convergence, in general after ≈ 30,000 to 80,000 steps, and on the hardware available. We used a desktop computer with 256 GB of RAM, 24 Intel® Xeon® W-2265 CPUs @ 3.50GHz, and NVIDIA® Corporation TU102GL Quadro RTX 6000/8000 graphics, running Ubuntu® 20.04.

### Declarations

In the article, use of trade, product, or firm names is for descriptive purposes only and does not imply endorsement by the authors or their Institutions.

## Supplementary information

Supplementary Information

## Data Availability

Landslide data used in the study are available from Zenodo at the following 10.5281/zenodo.7646106. Original rainfall data are the property of the individual regional governments of Italy and should be obtained from them. Rainfall duration and hourly cumulative rainfall for the rainfall events used in the study can be obtained from the authors upon reasonable request.

## Code availability

The software code used for the experiment is available upon request to the lead author.
