# Supplementary material for: Deep learning forecast of rainfall-induced shallow landslides
Source: Nat Commun. 2023 Apr 28;14:2466. doi: 10.1038/s41467-023-38135-y (PMC10147618; doi:10.1038/s41467-023-38135-y)
Supplement: Supplementary file 1 — Supplementary Information [file 41467_2023_38135_MOESM1_ESM.pdf]

# Supplementary information for: Deep learning forecast of rainfall-induced shallow landslides

Alessandro C. Mondini[1,2,⋆], Fausto Guzzetti[1,3], and Massimo Melillo[1]

[1] *Consiglio Nazionale delle Ricerche, Istituto di Ricerca per la Protezione Idrogeologica, via Madonna Alta 126, I-06128 Perugia, Italy*
[2] *Consiglio Nazionale delle Ricerche, Istituto di Matematica Applicata e Tecnologie Informatiche "Enrico Magenes", via de Marini 6, I-16149 Genova, Italy*
[3] *Presidenza del Consiglio dei Ministri, Dipartimento della Protezione Civile, via Vitorchiano 2-4, I-00189 Rome, Italy*
[⋆] *Corresponding author e.mail address: alessandro.mondini@ge.imati.cnr.it*

# Rainfall events and rainfall variables

To extract from the rainfall record of the individual rainfall events the rainfall variables $D_a$, $E_a$, $D_o = D_\ell$, and $E_o$, we used the procedure described in the Modelling datasets and rainfall variables subsection of the Methods section, in three steps, for rainfall events with landslides, and for rainfall events without landslides. Supplementary Figure 1 illustrates the subdivisions of the rainfall events considered in the work for rainfall events (a) without and (b) with landslides.

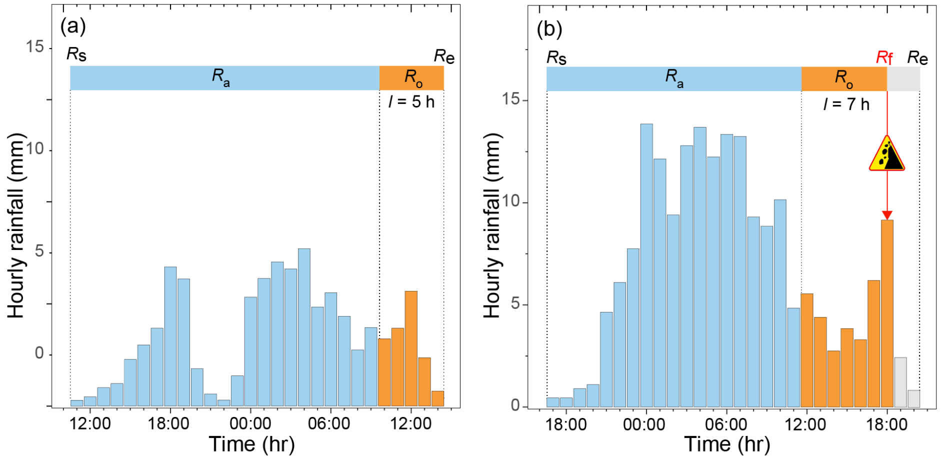


Supplementary Figure 1: Exemplification of rainfall events. (a) events without and (b) with landslides.

A rainfall event starts at the beginning of the event, $R_s$ and ends (a) at the end of the event, $R_e$ for rainfall events without landslides, or (b) at the time of the landslide occurrence, $R_f$ for rainfall events with landslides. In the figure, colours and lettering refer to Step 1 for (a) and Step 4 (b) of the procedure described in the Modelling datasets and rainfall variables subsection of the Methods section. Specifically, (i) blue bars show rainfall antecedent period, $R_a = [R_s; R_{f-\ell}]$, where $\ell$ is the lag; (ii) orange bars show rainfall triggering period, $R_o = D_\ell$, exemplified for $\ell = 5$ hr in (a) and for $\ell = 7$ hr in (b); and (iii) grey bars show rainfall period after the landslide occurrence, $(R_f; R_e]$ not considered in the analysis because irrelevant for landslide initiation. Square brackets indicate that the $R_s$ and $R_e$ times are included, and round bracket indicates that the $R_f$ time is excluded.

# Training, validation, and testing results

To prepare the set $\mathcal{O}$ of 2400 forecasting models, we adopt a train – valid – test data segmentation scheme [1] with bagging ensemble [2]. Supplementary Figure 2 shows the performances for three models in the train – valid (upper row), and the test (lower row) phases. Models shown were selected randomly from the pools of 100 models prepared for lags $\ell_1$, $\ell_{12}$, and $\ell_{24}$. Plots (a), (b) and (c) show the almost identical model performances for the train (blue) and the valid (orange) phases, and no (a) or very little (b, c) overfitting. Plots (d), (e) and (f) show the Receiver Operating Characteristic (ROC) curves [3] obtained in the test phase for the same three models. Pink dots show best trade off between model specificity and model sensitivity, and dotted lines show "no skill" predictions. The overall performance of the models on very unbalanced record sets is high, and very far from the "no skill" predictions (dotted lines in Supplementary Figure d,e,f).

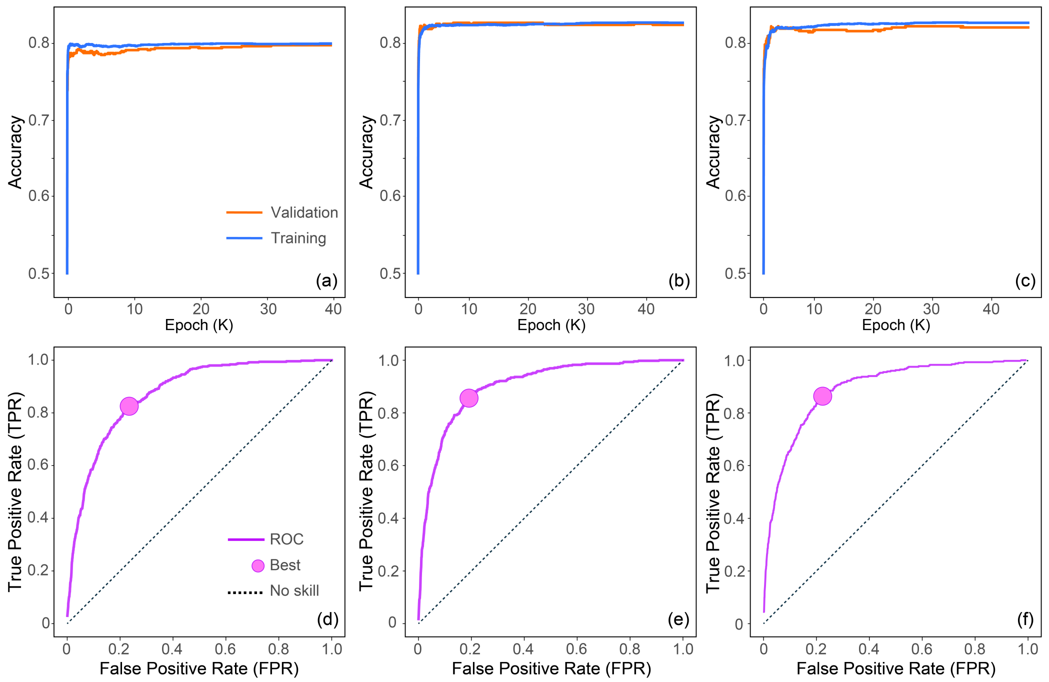


Supplementary Figure 2: Model performance examples. Training, validation, and testing (train – valid – test) results for models $\mathcal{M}^1_{37}$ (a, d), $\mathcal{M}^{12}_1$ (b, e), and $\mathcal{M}^{24}_{75}$ (c, f).

## Supplementary table 1: list of variables and symbols

| Variable, symbol | Description |
|---|---|
| $a_n^k$ | Activation of the $i$ neuron in the $k$ layer |
| argmax | The arguments of the maxima function |
| h | Sigmoid activation function |
| tanh | Hyperbolic tangent activation function |
| $c$ | Outcome of $S$, susceptibility value |
| $\kappa$ | Cohen's kappa coefficient |
| $\ell$ | Lag, a continuous sequence of hours, from 1 to 24 hours |
| $A_{ROC}$ | Area under the ROC curve |
| BA | Balanced Accuracy |
| $D$ | Duration of a rainfall event, $D = [R_s; R_e]$, in hours |
| $D_a$ | Duration of antecedent rainfall period, $D = [R_s; R_{e-L}]$, in hours |
| $D_o$ | Duration of triggering rainfall period, $D_o = D_L$, in hours |
| $E$ | Cumulated event rainfall, in mm |
| $E_a$ | Cumulated rainfall in the antecedent rainfall period, in mm |
| $E_o$ | Cumulated rainfall in the triggering rainfall period, in mm |
| $F$ | Landslide occurrence |
| $F_1$ | $F_1$-score statistics |
| $K$ | Epoch |
| $P$ | Probability |
| $p$ | Value of $P$ |
| $P(L\|R)$ | Probability of landslide occurrence, given a rainfall event, $R$ |
| $R$ | Rainfall event |
| $R_a$ | Rainfall event antecedent period |
| $R_f$ | Time of the landslide in a rainfall event, $R$ |
| $R_o$ | Rainfall triggering period |
| $S$ | Landslide susceptibility |
| $\hat{V}$ | Aggregated vote |
| $\mathcal{B}$ | Model bagging ensemble of 100 models, $\mathcal{M}$ |
| $\mathcal{M}$ | Model |
| $\mathcal{O}$ | Model set of 24 model bagging ensembles $\mathcal{B}$, or 2400 $\mathcal{M}$ |
| $\mathsf{T}$ | Subset of rainfall data points or rainfall records for model training |
| $\mathsf{V}$ | Subset of rainfall data points or rainfall records for model validation |
| $\mathsf{W}$ | Subset of rainfall data points or rainfall records for model testing |
| $\mathsf{X}$ | Set of rainfall data points not associated to a landslide |
| $\mathsf{Y}$ | Set of rainfall data points associated to a landslide |
| $\mathsf{Z}$ | Subset of rainfall events for independent model testing |
| $\beta$ | Bias in a neuronal connection |
| $\beta_{1,2}$ | ADAM optimization algorithm parameters |
| $\delta$ | Weight decay |
| $\acute{\epsilon}$ | ADAM optimization algorithm parameter |
| $\gamma$ | Dropout |
| $\mu$ | Mean, of normal distribution |
| $\sigma$ | Standard deviation, of normal distribution |
| $\tau$ | Transpose |
| $\theta$ | Single weight in a neuronal connection |

## Supplementary table 2: list of abbreviations

| Abbreviation | Description |
| --- | --- |
| hr | Hour |
| ECDF | Empirical Cumulative Distribution Function |
| FN | False negative |
| FP | False positive |
| FPR | False positive rate |
| KDE | Kernel density estimation |
| LSTM | Long-short term memory |
| ROC | Receiver operating characteristic curve |
| TN | True negative |
| TP | True positive |
| TPR | True positive rate |
| SVM | Support Vector Machine |

# Supplementary References


[1] Kuhn, M. & Johnson, K. Applied Predictive Modeling (Springer, 2013).

[2] Breiman, L. Bagging predictors. Machine Learning **24**, 123–140 (1996).

[3] Haixiang, G. et al. Learning from class-imbalanced data: Review of methods and applications. Expert Systems with Applications **73**, 220–239 (2017).
